# A Paper on Diphtheria

**Published:** 1861

**Authors:** 


A Paper on Diptheria.—Read before the New York Academy of Medicine, in June, 1861—By James Wynne, M. D., &c., &c.

This is a neatly executed monograph of 32 pages, published by Bailliere Brothers, 440 Broadway. It contains a very interesting history of Diptheria, with a fair summary of the prevalent views concerning its causes, pathology and treatment. It is well worthy of perusal and preservation.

